# The Treatment of Synovitis of the Knee-Joint

**Published:** 1897-05-29

**Authors:** E. Percy Paton

**Affiliations:** Surgical Registrar, Westminster Hospital, &c.


### THE TREATMENT OF SYNOVITIS OF THE KNEE-JOINT.

By E. Percy Paton, M.D., M.S., F.R.C.S., Surgical Registrar, Westminster Hospital, &c.

Synovitis of the knee-joint is so common, and yet in many cases so very obstinate to treatment, that a detailed consideration of the many means which may be used for its cure, whether occurring in an acute, subacute, or chronic form, needs no apology.

First, as to the acute form. Here, as, indeed, in all the varieties, it is most important, if possible, to find the cause, which is most commonly traumatism. If, however, no injury has occurred, cold may be responsible, or there may be a history pointing to a loose body or cartilage in the joint; but in any case, acute rheumatism must be excluded, as this is not amenable to merely local means such as should be instituted for the ordinary traumatic form.

The first essential of treatment is rest, and this should be attained as far as possible by placing the joint in the most comfortable position. What is usually done is to put the leg and thigh upon a back splint in a fully extended position. This, however, is by no means altogether satisfactory; for one thing, it is anything but a comfortable position for the joint to be kept fully extended, and as in synovitis there need be no fear of permanent deformity of the joint by destruction of its ligaments and cartilages, there is no objection to putting it up in a slightly flexed position, which will usually be found to be much more comfortable than full extension. This can be very conveniently done by taking a large pillow, making something of a trough in its centre by manipulating the feathers to either side, and then laying the leg from thigh to foot in this hollow, and tying both pillow and leg round in several places with some broad pieces of bandage. An additional advantage of this method is that it requires no padding or splints, as a pillow is always handy, and can be obtained anywhere. After rest has thus been instituted, cold should be applied either by an ice-bag or evaporating lotion, in the latter care being taken to keep the lint constantly wetted. Should the effusion be excessive, recovery may be accelerated very materially by removing the fluid either with a trocar and cannula or by aspiration; and this will be of especial advantage if any of the effusion be blood, as is not infrequently the case after severe injury. This blood, if left, is likely to leave fibrinous masses in the joint, and impair its movements subsequently; it is, however, sometimes difficult to get it to run through a hollow needle. Of course, if the fluid be drawn off, every aseptic precaution must be taken, or much damage may be done. Treatment by rest and cold must not be kept up too long, and must be dispensed with as soon as all signs of active inflammation are gone, as it is then a disadvantage to reduce the circulation by cold, which is now required to be as active as possible about the joint to get rid of all excess of fluid. Absorption of fluid is best promoted by the use of skilfully-applied massage, both over and above and below the joint, accompanied by, at first, passive movement, and then active. To this method of treatment almost all acute cases of synovitis will yield with great rapidity. Certain cases, however, show a very great tendency to recur at the very slightest provocation. This is sometimes due to an adhesion in the joint which, when stretched by partial flexion, irritates the synovial membrane; and if it be broken down by fully flexing the joint, if necessary under gas, the trouble is at once cured; or the joint may be very easily strained, owing to the muscles and tendons around being poor in tone and power, thus giving it no protection. This trouble is especially helped by massage and exercise carefully carried out. Or, lastly, a loose body or cartilage may be present. If the former can be found it must be removed by direct incision over it into the joint; of course, aseptically. It is advisable to find and fix the body first before starting the operation, otherwise it may be subsequently difficult to find. It may, however, be easily fixed by transfixing it with a needle before the anæsthetic is given. A loose semilunar cartilage is not quite so simply treated. A good deal may be done by rest combined with massage, and exercises devised to strengthen the muscles round the joint, while avoiding those movements which cause the cartilage to become dislocated; or a well-made knee cap or truss arrangement may be worn to keep it in its place. If these measures are not satisfactory, operation may be undertaken either to suture the cartilage in its place, or to remove a part of it; but even this extensive interference with the joint is not always as satisfactory in its results as might be wished.

Unfortunately, a good many cases of synovitis which were at first acute do not settle down as easily as above described; but instead of the fluid being rapidly absorbed, the effusion to a large extent remains, showing but little tendency to clear up. Recourse must then be had to counter-irritation, and this is best applied in the form of blisters in the neighbourhood of the joint. It is usually best not to employ one large blister at first, but to put one of moderate size on first above the patella, then as that begins to heal to place one below, and then in succession one on each side. By this means counter-irritation can be kept up more continuously than by the application of one large blister. After the blisters have healed, and if the fluid be gone, the joint may be strapped with Scott's dressing (Unguentum Hydrargyri Co.) This is to be done by spreading the ointment on thin flexible leather, cut into strips about an inch broad and long enough to go one and a half times round the joint; these are then put on from below upwards, the one above slightly overlapping that below it; a bandage is then put on over all; by this means the advantage of the ointment is obtained, also the movement permitted to the joint is considerably limited, so that the patient may be allowed to get up and about, using the limb in moderation. Sometimes even blistering as above described does not remove the fluid. Should this be the case counter-irritation by the actual cautery will sometimes be found to succeed. As the process is naturally a painful one, an anæsthetic should be given, and then, using a Paquelin's cautery at a dull red heat, several lines about half an inch broad are burnt by drawing the point of the hot iron across the front of the joint, either vertically or horizontally. These burns should not go through the true skin, and may be subsequently dressed with any simple ointment, and the joint kept at rest until they are healed. When the fluid has gone then massage, as described above, may be used with the greatest possible advantage, and in fact some cases, even when fluid is still present, improve remarkably where it is systematically and carefully carried out; while, as the case improves, it can be combined with passive movement, insuring the freedom of the joint from any adhesions.

Even in spite of counter-irritation and other treatment, as above described, a few cases of synovial effusion still refuse to clear up, the joint still remaining full of fluid often in considerable quantity, but showing practically no other sign of inflammatory trouble. In some of these cases tubercle is really the basis of the mischief. Into the treatment of these cases it is not proposed to enter here, but in many the condition present is merely a hydrops of the joint, which, however, tends to greatly damage its utility by considerably stretching both capsule and ligaments. It is best now to first empty the joint by aseptic aspiration, and then to attempt to prevent a recurrence of the fluid by keeping up moderate continual pressure by use of an elastic Martin's bandage. At first the knee must be kept at rest, but later the patient may be gradually allowed to use it while still wearing the bandage. Should the fluid still recur and refuse to be reabsorbed, it may be again drawn off with a trocar and cannula, and then one to two drams of tincture of iodine may be injected through the cannula, to try and alter the secreting power of the synovial membrane just as in the treatment of an ordinary hydrocele. When the irritation which this causes has subsided treatment by the elastic bandage can be continued.

Should even this fail, drainage of the joint for a time by means of two openings, one on either side of the patella, and a tube passed right through may be tried. After the fluid has been let out and the tube put in, the joint is put up in a splint with a permanent dressing, which need not be disturbed, if all goes well, for ten days or a fortnight, when the tube can be removed and the wounds allowed to close, the joint being then first moved passively and gradually allowed to be used, supported by an elastic bandage. One of the advantages of this method is that it allows the interior of the joint to be examined, and so tubercle, as a possible cause of the obstinacy of the case, can be excluded. Should even this fail, it is best to put up the knee in an extended position, and keep it so for a prolonged period. This is most easily and comfortably done by the use of a leather splint, lined with wash-leather and laced up the front. It is conveniently made more rigid by having rivetted to it behind a bar of iron, the lower end of which it is sometimes convenient to have prolonged beyond the leather splint, so that it can be fitted into a slot in the heel of the boot, and thus prevent the whole splint from slipping down the leg. By the use of this splint continual pressure can be kept up along with absolute rigidity, and yet the patient can be allowed to get about.

No small part of the treatment of synovitis of the knee, especially when it becomes chronic, consists in improving the general condition of the patient by placing him under the best possible hygienic circumstances, and ensuring that plenty of fresh air is obtained even when exercise cannot be permitted; a change of air to the country, or better still to the seaside, being often very beneficial, while the lassitude and want of appetite produced by being confined to the house in a patient who previously has often been accustomed to abundant open-air exercise, cannot but be prejudicial to the local as well as the general condition.

